# Transparent Polymer Opal Thin Films with Intense UV Structural Color

**DOI:** 10.3390/molecules27123774

**Published:** 2022-06-11

**Authors:** Giselle Rosetta, Matthew Gunn, John J. Tomes, Mike Butters, Jens Pieschel, Frank Hartmann, Markus Gallei, Chris E. Finlayson

**Affiliations:** 1 Department of Physics, Prifysgol Aberystwyth University, Aberystwyth SY23 3BZ, UK; grm4@aber.ac.uk (G.R.); mmg@aber.ac.uk (M.G.); jjt12@aber.ac.uk (J.J.T.); 2 Varichem Co., Ltd., Brynmawr NP23 4BX, UK; 3 Minton Treharne and Davies, Cardiff CF14 7HY, UK; mike.butters@minton.co.uk; 4 Polymer Chemistry, Saarland University, Campus C4 2, 66123 Saarbrücken, Germany; jens-michel.pieschel@uni-saarland.de (J.P.); frank.hartmann@uni-saarland.de (F.H.); markus.gallei@uni-saarland.de (M.G.); 5 Saarene, Saarland Center for Energy Materials and Sustainability, Campus Saarbrücken C4 2, 66123 Saarbrücken, Germany

**Keywords:** photonic crystals, UV spectroscopy, optical characterization, polymers, thin film coatings, UV reflecting films

## Abstract

We report on shear-ordered polymer photonic crystals demonstrating intense structural color with a photonic bandgap at 270 nm. Our work examines this UV structural color, originating from a low refractive index contrast polymer composite system as a function of the viewing angle. We report extensive characterization of the angle-dependent nature of this color in the form of ‘scattering cones’, which showed strong reflectivity in the 275–315 nm range. The viewing range of the scattering was fully quantified for a number of planes and angles, and we additionally discuss the unique spectral anisotropy observed in these structures. Such films could serve as low-cost UV reflection coatings with applications in photovoltaics due to the fact of their non-photobleaching and robust mechanical behavior in addition to their favorable optical properties.

## 1. Introduction

Photonic crystals have been a topic of intense interest in optics and photonics since Yablonovitch [1] and others [2,3,4,5] first reported on them as the optical equivalent of semiconductors. These materials, well-ordered arrays of modulating refractive index, demonstrate photonic bandgaps that can be engineered to prevent the propagation of light bands [6]. As a result of the constructive diffraction of reflected wavelengths, photonic crystals display a brilliant iridescent structural color that varies as a function of viewing angle. This phenomenon appears throughout the natural world in butterflies [7,8], beetles [9], and berries [10].

Photonic crystals demonstrating ultraviolet structural color have been documented extensively. However, the overwhelming majority of these reports pertain to examples in nature (such as feathers) [11,12,13] as opposed to synthetic systems. Furthermore, there is a lack of extensive optical characterization and systematic engineering of such structures, and, as such, very little understanding thereof. This is despite the significant potential of photonic crystals operating in the UV regime, with a focal point of interest being their application as coatings to prevent photodegradation, for example, in dye-sensitized solar cells. Cai et al. [14] reported on the benefits of silica-templated polymer inverse opal coatings on the performance of dye-sensitized solar cells, with the application of the UV-shielding photonic crystal coating resulting in a slower photochemical degradation of the solar cell than when the cell was uncoated. Notwithstanding the broad interest in UV reflecting transparent films [15,16], there are relatively few reports of photonic crystals based on low-refractive index soft-matter material approaches for UV structural color.

It is with this in mind that polymeric materials present a refreshing paradigm in photonic crystals. In contrast to often brittle inorganic sedimentary opals, which when freestanding are only microns or millimeters squared in terms of area [17,18], polymer photonic crystals are mechanically robust [19,20] and give rise to a range of unique structural coloration phenomena such as strain tunability [21,22]. On account of their favorable rheological properties, there is an additionally strong material compatibility with roll-to-roll and shear ordering techniques [23,24], which are highly conducive to generating structures with area 10s of meters squared at low cost. Here, we report on polymer opals with intense UV structural color and discuss the detailed characterization of the reflectivity properties of these engineered nanostructures.

**Figure 1:**
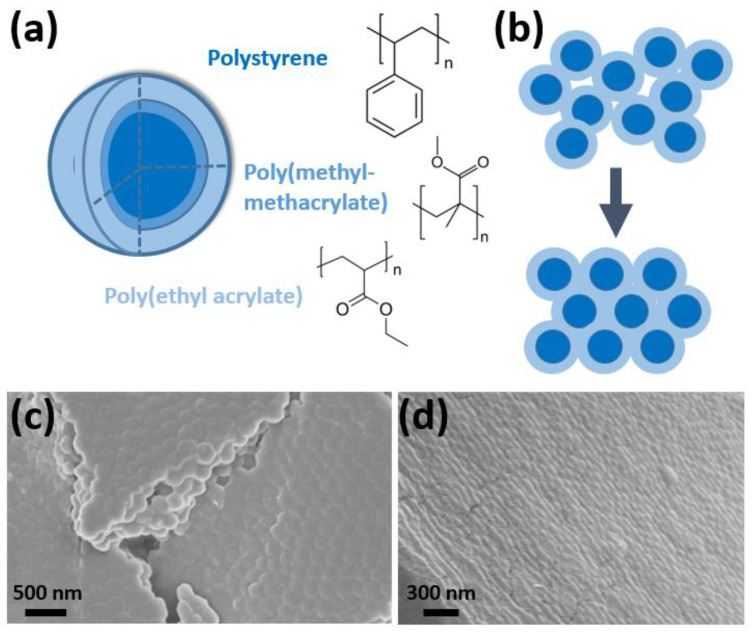
(**a**) The polymer opal core–shell particle, consisting of a polystyrene core, poly(methyl-methacrylate) grafting interlayer, and the poly(ethyl acrylate) shell, with the chemical structures of each layer shown. (**b**) The shear-assembly of these particles into random-hcp crystalline arrays is illustrated, with the interstices between PS cores filled by the grafted PEA matrix. The ordered surface of a 5 BIOS opal was imaged by scanning electron microscopy (SEM), as shown in (**c**), and (**d**) shows how the hexagonal close packing permeated the depth of the film after 40 BIOS passes. Changes in microporosity and homogeneity are a byproduct of the film preparation techniques for microscopy.

Polymer opals [25,26,27] are crystalline arrays of core–shell nanoparticles of diameters ranging between 100 and 300 nm. These particles are synthesized by emulsion polymerization, and adjustment of the reaction parameters allows for tuning of the particle size thereby providing access to the desired structural color properties. The core–interlayer–shell (CIS) particles are composed of a polystyrene (PS) core, poly(methyl-methacrylate) (PMMA) grafting interlayer, and a poly(ethyl acrylate) (PEA) shell [28] as shown in Figure 1a. These particles are melt-extruded into ribbons, during which the outer shell is melted to fill the interstitials surrounding the cores, which remain grafted in position by the PMMA interlayer. Lamination of these ribbons between PET films and subsequent shearing about heated rollers serves to induce order in the structure, as illustrated in Figure 1b. This process is known as bending-induced oscillatory shear (BIOS), previously reported by Zhao et al. [24]. Following BIOS processing, the polymer opals can then be readily removed from the PET, resulting in large area, freestanding, well-ordered films, displaying brilliant structural color originating from a small refractive index contrast of only approximately Δn ≈ 0.11 [28]. Fabrication of the thin films is discussed further in Section 3. 

Polymer opals displaying visible structural color have been characterized extensively [29,30,31,32,33], with the tunability of the structural color with sphere geometry and size being particularly well documented. However, we believe our work to be among the first reports of these systems being engineered toward the ultraviolet band of the electromagnetic spectrum, thus forming the primary focus of this paper. We report on detailed angle tuning with incidence and viewing angles, using a bespoke goniophotometry apparatus, as well as transmittance characterization demonstrating favorable transmissibility across the visible spectrum.

## 2. Results

### 2.1. Structural Characterization

**Figure A1:**
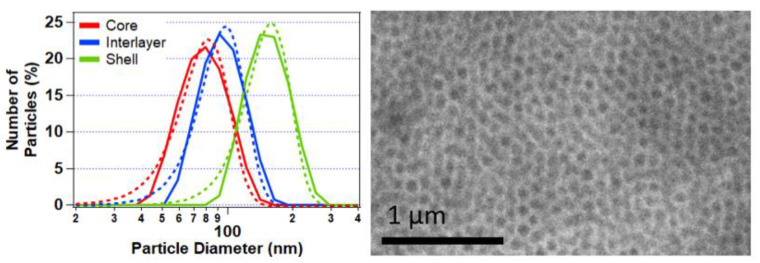
DLS data (**left**) showing the distribution of the nanoparticles following growth of the core, interlayer, and shell, with the Gaussian fits denoted by the dashed lines of the respective color. The *y*-axis denotes the percentage of the total number of particles of each size contributing to the total scattering intensity. A reproducibly low polydispersity index (PDI) of approximately 0.006 was obtained for the final CIS particles, of a diameter of 151 nm. This was calculated by Malvern Zetasizer proprietary software as $PDI={\left(standard\;deviation/mean\right)}^{2}$ [37]. TEM of a 40 BIOS opal after microtoming showing random hexagonal close packing (**right**).

**Figure A2:**
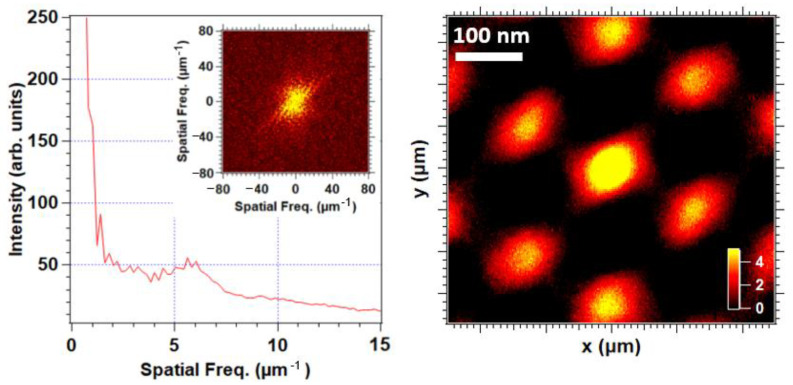
Fast Fourier transform (FFT) analysis (inset) of the SEM, as shown in Figure 1c of the 5 BIOS film. Examination of the corresponding radial distribution in the (**left**) panel shows a single predominant nonzero spatial frequency, with a peak intensity at approximately 6 μm^−1^, corresponding to a real-space periodicity of approximately 165 nm in good agreement with the DLS characterization of the particle size (and, thus, the 111-plane packing distance). The nature of this packing was additionally elucidated in terms of a 2D spatial autocorrelation function (**right**), showing a distinctive six-point symmetry indicative of hexagonal close packing.

An example of BIOS ordering is shown in Figure 1c, whereby the SEM of a five BIOS opal showed consistent low-defect hcp packing over domains of several particle lengths on the sample surface. Ordering was also seen to have propagated a few particle layers. For a 40 BIOS opal, as shown in Figure 1d, the hcp packing had fully permeated the depth of the sample over a length of several microns. Further TEM imaging of our shear-ordered films is given in the Appendix A (Figure A1), in addition to a more detailed DLS characterization of the particle shell size and distribution. Corresponding image analysis is reported on in Figure A2, which probes the structural features of the correlation length and packing quality. This work is in excellent agreement with the many previous structural studies on polymer opals as reported elsewhere [25,29,34].

### 2.2. Transmittance Characterization

Polymer opal films were fabricated from polystyrene cores of 91 nm diameter with a total core–shell diameter of 155 nm. The DLS characterization of these particles is given in Appendix A with further micrograph imaging. Varying levels of order were then induced in the films using BIOS shear ordering techniques [24] as discussed in Section 3. Because ordering has previously been found to reach an optimized state after approximately 40 BIOS passes, we report on opals with 0, 10, 20, and 40 passes.

**Figure 2:**
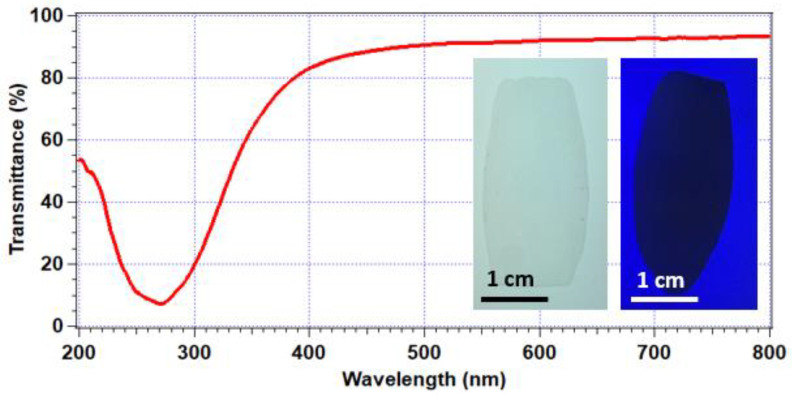
Normal incidence transmission of a well-ordered polymer opal with a photonic bandgap at 270 nm; inset L: polymer opal coated onto a white-light box, demonstrating high transparency; R: polymer opal coated onto paper and illuminated by UV LED, whereby the photonic bandgap prevented fluorescent excitation, resulting in a shadow.

Figure 2 shows the measured transmission spectrum of a maximally ordered opal at normal incidence. A clear photonic bandgap is shown at 270 nm, where transmission was reduced significantly to approximately 7%. As an important consistency check and to verify the origins of the bandgap effect, we were readily able to infer the lattice parameter and interlayer spacing of the r-hcp lattices formed based on the direct measurements of particle size (DLS/TEM) as presented in Appendix A. The Bragg–Snell law at normal incidence then accurately predicts the resonant reflectivity wavelength (*λ*):

(1) $$\lambda =2d_{hkl}n_{0},$$

where the effective refractive index is $n_{0}$ ≈ 1.52 (as measured with an independent refractometry method) and $d_{111}=a/\sqrt{3}$. Using the measured values of *a* = 155 nm, this gives *λ* = 272 nm, in close agreement with the experimental value of the maximum attenuation of transmitted light. 

In contrast, transmittance ranged in value between 78 and 93% across the visible regime. The transmittance values tending to 93% showed excellent agreement with Fresnel’s equations for transmission of light through consecutive air-dielectric and dielectric-air interfaces, with the average polymer opal refractive index taken as 1.52 [35]. Figure 2 shows the visual transparency of the polymer opal as viewed on a light-box illuminator (left inset). Conversely, the right inset in Figure 2 illustrates the effect of the ultraviolet bandgap; the opal was placed on fluorescent paper and illuminated by a 275 nm LED. The photonic bandgap served to prevent transmission of the light and, thus, excitation; therefore, the opal appeared as a shadow while the substrate fluoresced.

### 2.3. Reflectance Characterization

**Figure 3:**
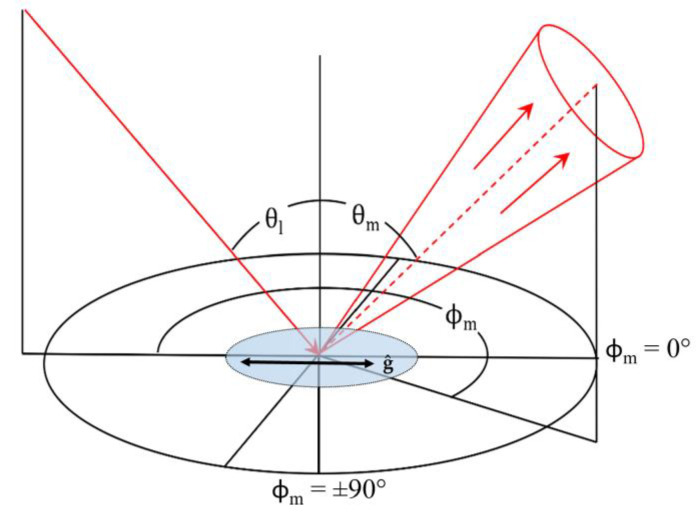
The coordinate system nomenclature of the custom goniophotometer. θ_I_ corresponds to the illumination angle; θ_m_ to the measurement (viewing) angle; ϕ_m_ to the azimuthal measurement angle as displacement from the plane of incidence. A polymer opal was placed at the center of the focused optics, with the direction of BIOS shear, **ĝ**, indicated.

Goniometric studies of opals of 0, 10, 20, and 40 BIOS passes were examined to study the effect of ordering on the structural color scattering cone. The spectral reflectivity of the opals was measured across the hemisphere of viewing in the plane of incidence, where the opal was aligned such that the direction of the shear was parallel to the incidence. The geometry of the measurement setup, relative to the BIOS direction vector **ĝ**, with the scattering angles labeled is shown in Figure 3.

**Figure 4:**
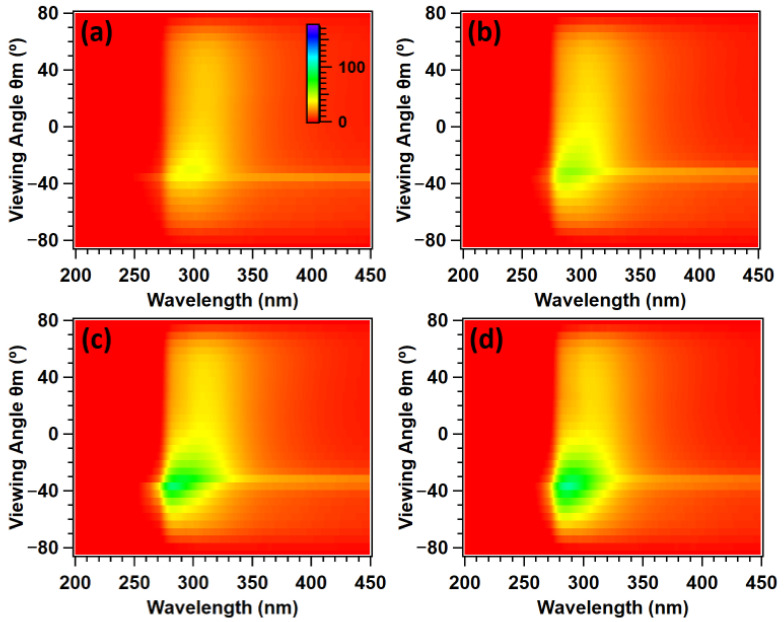
Scattering cones of (**a**) 0; (**b**) 10; (**c**) 20; (**d**) 40 BIOS passes. Specular reflection and obscuration artifacts were removed, and the viewing angle is in the plane of illumination (ϕ_m_ = 0°). The *z*-axis corresponds to the reflectance (%), with scale inset (**a**).

These results are presented in Figure 4, which show samples of gradually improving order. Spectra were taken across viewing hemispheres in 5° increments and spliced together to form an intensity matrix. Figure 4a denotes a 0 BIOS pass opal with only superficial surface ordering from the PET lamination process, and Figure 4d shows a polymer opal with a bulk ordering far surpassing the Bragg penetration depth. The *z*-axis denotes the measured reflectance (%), with the common scale inset in Figure 4a. Obscuration and specular reflection effects have mostly been corrected for in these figures, although some remnants of specular reflection remain, as removal was limited by the measurement resolution.

There were two main characteristic features of these spectra. Firstly, the ‘tail’-like scattering behavior, visible across the measured hemisphere ranging from approximately 280 to 330 nm. This was shown to grow in intensity with successive ordering. From Figure 2a, one can see that this clearly corresponded to structural color. However, from examination of the intensity at each viewing angle for each sample shown in Figure 4, this coloration was also shown to be omnidirectional and thereby non-iridescent in nature. This is hence likely to originate from resonant Mie scattering [36].

The primary scattering feature of interest, however, was the main structural color scattering cone. One can observe some faint coloration for even 0 BIOS passes due to the superficial surface layer ordering. This becomes more intense for 10 BIOS (Figure 4b), and at 20 BIOS (Figure 4c), one can clearly observe this coloration, characterized by intense scattering over the 275–325 nm band for select viewing angles. This structural color becomes highly intense for 40 BIOS passes (Figure 4d), and the scattering cone was significantly widened with an angular full width half maximum (FWHM) of 32.5° as described in Table 1. We previously reported on the change in spatial frequency of the constituent nanoparticle arrays using Lomb periodogram Fourier transform methods and determined the sharpness of the distribution of these frequencies [29]. The change in the ratio of the distribution sharpness to frequency was a clear indicator of the improving ordering, which has previously [29] been seen to correlate with increases in the full width half maximum of the structural color scattering cone.

These results also suggest an exponential plateau on the angular width of the scattering cone with increasing numbers of shear passes applied as previously noted for polymer opals with visible structural color [29,30]. The cone with a 16.2° width for 10 BIOS was broadened by 9.9° for a further 10 BIOS passes, which was broadened by only 6.4° for an additional 20 BIOS passes. These data imply that further angular broadening post-40 BIOS should not be discounted, and this is a topic for deeper investigation.

**Figure 5:**
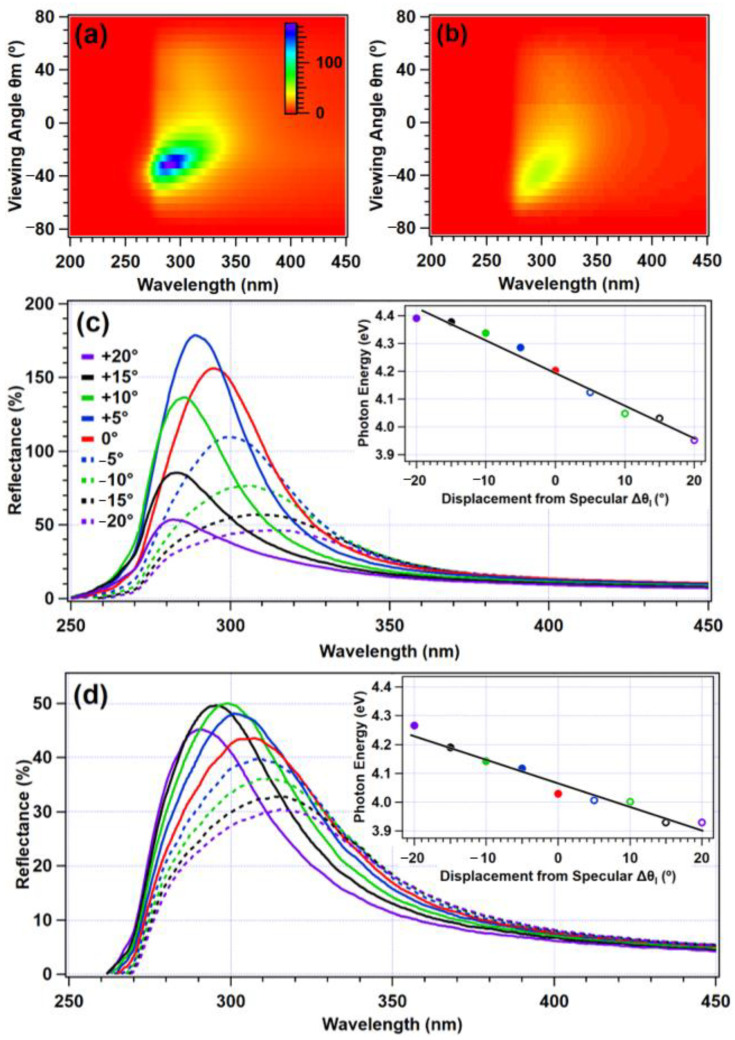
Scattering cones of a 40-shear pass polymer opal, illuminated at 30° from the zenith for (**a**) ϕ_m_ = 30° and (**b**) ϕ_m_ = 60°, with reflectance on the *z*-axis with scale inset in (**a**). The reflectance spectra for viewing angles between 20° above and 20° below the specular direction in 5° increments are shown in (**c**) for the ϕ_m_ = 30° orientation and in (**d**) for ϕ_m_ = 60°. The wavelengths of the peak intensity for each viewing angle were extracted and are displayed (insets) in terms of photon energy, with the dots and hoops corresponding to the solid and dashed lines, respectively.

The characteristic anisotropy, shown in Figure 4, became especially clear when examined outside of the specular direction and plane-of-incidence. Figure 5a,b show scattering cones for an opal of 40 BIOS passes, illuminated at 30° from the zenith parallel to the shear direction, viewed in the ϕ_m_ = 60° and 30° orientations. Figure 5a shows how in comparison to viewing in the plane of incidence (Figure 4d), there was appreciably greater spectral anisotropy. This persisted for as far as 60° displaced from the plane of incidence, as shown in Figure 5b, where asymmetry in the viewing remained significant on either side of the specular angle. This figure also demonstrates that there was a broad angular spread of the reflectance characteristics of the structural color, with a moderate intensity even when widely removed from the specular angle.

Figure 5c,d show, in further detail, how the tuning of the structural color changed in both azimuthal planes ϕ_m_ = 30° and 60° as a function of the viewing angle. This is shown over the 40° section of the viewing hemisphere around the specular direction (θ_m_ = 30°), with colors corresponding to the same angular displacement on either side of θ_m_ = 30°. These figures clearly show variation in the reflected intensities on either side of the specular direction. Reflectance was seen to notionally exceed 100% in places, because the Lambertian calibration standard scatters light omnidirectionally, whereas the opaline photonic crystals essentially act as concentrators by comparison because of the preferential scattering. The wavelengths corresponding to the peak intensities of each viewing angle were extracted and converted to photon energy. This is shown in the insets for Figure 5c,d, demonstrating a linear relationship between the most intensely reflected photon energy and viewing angle. This angular tuning was thus consistent with the Bragg–Snell law. The spectral anisotropy is quantified in additional detail in Table 2, again showing the viewing angle in terms of displacement from the specular direction θ_m_ = 30°. Peak wavelengths were reported for the same viewing range as previously examined: 20° on either side of θ_m_ = 30°. In the plane of illumination, the wavelength tuning Δλ = 32 nm. While this roughly corresponded with a Δλ of 31 nm for ϕ_m_ = 30°, Δλ decreased to 25 nm for ϕ_m_ = 60°. This clearly demonstrates that the wavelength range of the structural color decreased as a function of the angular displacement from the specular plane. Furthermore, for the ϕ_m_ = 90° direction (orthogonal to the plane of incidence), the wavelength tuning became negligible (Δλ = 2 nm).

## 3. Materials and Methods

### 3.1. Synthesis of CIS Particles

The polymer opal nanoparticles were fabricated by stave-fed multistage emulsion polymerization [34]. A diagram of the CIS particle layers is shown with the main material component in Figure 1a. Emulsifier quantities were varied beyond previously reported methods to obtain the desired particle size, and 1.12 L of deionized water, 14.4 g styrene, 1.6 g 1–4, butanediol-diacrylate (BDDA), and 1.17 g sodium dodecylsulfate (SDS) were stirred in a round-bottomed flask at a rate of 200 rpm and maintained at a temperature of 65–70 °C throughout; 12 mL deionized water, 2.07 g sodium persulfate, and 0.29 g sodium bisulfate were then added to initiate the reaction. After 10 min, 360 mL deionized water, 280 g styrene, 0.97 g SDS, 1.6 g potassium hydroxide, and 0.88 g Dowfax 2A1 50% surfactant were added dropwise at a rate of 4 g/min. This solution was reacted for 30 min, forming the polystyrene cores. Half of the solution was then removed. One milliliter of deionized water and 0.05 g sodium persulfate were added to reinitiate the reaction to allow for the growth of the interlayer; 64 mL deionized water, 50 g ethyl acrylate, 0.6 g allyl methacrylate, 0.1 g SDS, and 0.42 g Dowfax 2A1 50% were added dropwise at a rate of 4 g/min. After 30 min, 320 mL deionized water, 201.1 g ethyl acrylate, 70 g iso-butyl methacrylate, 8.4 g 2-hydroxyethyl methacrylate, 0.85 g SDS, and 0.4 g potassium hydroxide were added in order to grow the particle shell. The reaction was stirred for an additional 15 min, after which the solution was cooled. The solution was added to a bath of methanol (2 L) and saturated brine (20 mL), which allowed the particles to deposit as sediment. The solvent was removed, and the isolated particulate matter was dried by fluid bed.

Particle growth was monitored at 30 min intervals with dynamic light scattering (DLS). This was performed using the Malvern Nano ZS Zetasizer. The nanoparticle solution was diluted in deionized water at a 1:250 mL ratio. For each sample, this was averaged over approximately 15 times per run, and the averages of three runs are reported for each particle layer. The resultant particles had core, interlayer, and shell diameters of 91, 112, and 155 nm, respectively, with a final polydispersity index of 0.006. Further details of the PDI definition and calculation are given in Appendix A.

### 3.2. Bending-Induced Oscillatory Shear (BIOS)

Following drying, the nanoparticles were homogenized and melt-extruded, using the Haake minilab co-rotating twin extruder at 150 °C, into ribbons of approximately 1 mm in thickness. These ribbons were subsequently laminated between PET sheets by two aluminum rollers preheated to 80 °C. The PET was precleaned with isopropanol and ethanol to ensure optimal material adhesion. This procedure results in a ‘Timoshenko sandwich’, with a polymer opal with approximately 150 µm in thickness between the PET sheets. The sandwich beam was then drawn over rollers 2 cm in diameter and heated to 85 °C at a shear rate of approximately 1 cm/s. This was repeated for a number of shear passes between 10 and 40. This initially created ordering beginning at the surfaces of the sample, which subsequently permeated inwards with further shear, and a basic representation of this process is shown in Figure 1b. The physical mechanisms of the BIOS process shear ordering are theoretically elucidated in detail elsewhere [24,31].

### 3.3. Electron Microscopy Imaging

Scanning electron microscopy (SEM), as shown in Figure 1c,d, was carried out on a GeminiSEM 500 Zeiss Sigma VP device with SmartSEM Version 6.07 software. The opal films were mounted on an aluminum stud using adhesive copper tape and sputter-coated with approximately 6 nm of platinum using an Automatic Turbo Coater PLASMATOOL 125 SIN 2020_131 (Ingenieurbüro Peter Liebscher). Transmission electron microscopy (TEM) images were recorded on a JEOL JEM-2100 electron microscope at a 200 kV acceleration voltage via a Gatan Orius SC1000 camera in the bright field mode. Software processing was carried out using Gatan Microscopy Suite.

### 3.4. Transmittance and Reflectance Characterization

Spectroscopic measurements were made using two optical setups. In both cases, broadband light with a high UV content was provided by an Energetiq fiber-coupled laser-driven light source (EQ-99FC) with a 400 µm UV transmitting fiber. This was free space coupled with a Thorlabs UV-enhanced off-axis parabolic reflective collimator (RC08SMA-F01). Detection was made with a Stellarnet Silver Nova CCD spectrometer fitted with a 14 µm slit.

Transmission measurements were made by mounting a fiber collimator approximately 50 mm from the 9.5 mm port of an Ocean Optics 38 mm Spectralon-lined integrating sphere (FOIS-1). The integrating sphere served to reduce the sensitivity of the measurements to minor changes in the beam path caused by the introduction of the sample; thus, providing significantly more consistent measurements. The beam was blocked for a spectrometer dark signal measurement, and a light source reference measurement was taken with the sample removed from the beam path. For measurement, the sample was mounted between the fiber collimator and integrating sphere. The integration time was 3 ms, and 15 measurements were averaged to reduce noise. The transmittance of the sample was evaluated by the Stellarnet Spectrawiz software (v.5.33).

Bidirectional scattering measurements were made using a custom-made manual three-axis goniometer. The light source fiber collimator was mounted 400 mm from the sample on an arm attached to a rotation stage, so that the angle of incidence (θ_I_) could be adjusted about the sample plane. The light source provided approximately collimated light, which illuminated a circular spot approximately 15 mm in diameter on the sample at normal incidence. The scattered light was collected by a 25 mm diameter UV-enhanced aluminum-coated mirror at a distance of 260 mm from the sample, which focused the reflected light into the spectrometer-collecting fiber. The detection optics collected a cone of scattered light with a half angle of 2.8° and observed a circular area of the sample of approximately 5 mm in diameter at normal incidence. The detection optics were mounted on two perpendicular rotation stages so that the viewing angle (θ_m_) and azimuthal angles (ϕ_m_) could be adjusted independently. This instrumentation is illustrated in Figure 3, with the co-ordinate system nomenclature as indicated.

The detection optics were blocked to obtain spectrometer dark signal measurements prior to each set of acquisitions. A reference measurement was made on a Spectralon 99% white reflectance target illuminated at normal incidence and observed at 30° (to approximate Lambertian scatter). As the samples were highly transparent, they were mounted on acrylic substrates, which were coated with a black tape (approximately 7% reflectance) to minimize the scattering contribution from the sample mounting.

## 4. Conclusions

In conclusion, we reported on highly transparent polymer thin films displaying strong UV structural color in analogy with structurally ordered systems in nature. We investigated the ‘scattering cones’ of this coloration as a function of shear-order processing. For highly ordered opaline films, we reported on this color for a range of viewing planes and viewing angles. We showed how the angular width and intensity of the structural color increased with successive shear BIOS ordering. Our results show the angular width of the scattering in multiple planes of viewing, and how the observed structural color changed as the viewing angle moved outside the scattering cone, thus comprehensively demonstrating the angular tuning of the resonant energy/wavelength across the UV-A and UV-B optical bands. Our systems have a range of advantages over other films in that they are low cost, recyclable, non-photobleaching, and can be fabricated with solvent-free methods. In addition, as freestanding films with good adhesive properties, polymer opals are compatible with simple retroactive installation on a range of surfaces—removing the need for conventionally expensive and time-consuming deposition methods. We envision that the techniques we applied will, in the future, allow for fully tunable engineered polymer photonic thin films. Solar cell technology, in particular, would highly benefit from the application of transparent UV films as tools for extending the lifespan of dye-sensitized photovoltaic devices. We foresee this being the subject of highly promising future work, as we aim to apply our methods to bespoke engineering reflectance coatings in other pertinent regions of the nonvisible electromagnetic spectrum such as the near-infrared.

## Figures and Tables

**Table 1:** Full width half maxima of scattering cones as a function of BIOS passes, determined from a Gaussian fit of the maximum intensities at each measured angle for each sample. It was not possible to determine this value for 0 BIOS with accuracy. The peak intensity was recorded for these fits.

No. BIOS Passes	0	10	20	40
FWHM (°)	-	16.2 ± 2.3	26.1 ± 3.0	32.7 ± 2.5
Peak reflectance (%)	42.44 ± 3.5	76.1 ± 7.3	113.2 ± 11.6	103.9 ± 7.2

**Table 2:** The wavelengths corresponding to the maximum reflectance for viewing angles ±20° displaced from the specular direction of θ_m_ = 30° for a number of azimuthal viewing planes, for a 40 BIOS pass opal. Error in wavelengths of ±0.5 nm.

Displacement (°)	20	15	10	5	0	−5	−10	−15	−20
ϕ_m_ = 0°	282	283	286	289	295	300	306	307	314
ϕ_m_ = 30°	282	283	286	289	294	300	306	307	313
ϕ_m_ = 60°	290	296	299	301	307	309	310	315	315
ϕ_m_ = 90°	314	314	316	316	316	316	317	317	317

## Data Availability

Data can be accessed from the Aberystwyth University PURE repository, DOI: 10.20391/044206d3-08ce-4f34-8dfd-a88bf4c679bf.

## Sample Availability

Samples are available from the authors.
